# *Mx1, OAS1* and *OAS2* polymorphisms are associated with the severity of liver disease in HIV/HCV-coinfected patients: A cross-sectional study

**DOI:** 10.1038/srep41516

**Published:** 2017-01-31

**Authors:** Mónica García-Álvarez, Juan Berenguer, María A. Jiménez-Sousa, Daniel Pineda-Tenor, Teresa Aldámiz-Echevarria, Francisco Tejerina, Cristina Diez, Sonia Vázquez-Morón, Salvador Resino

**Affiliations:** 1 Unidad de Infección Viral e Inmunidad, Centro Nacional de Microbiología, Instituto de Salud Carlos III, Majadahonda, Madrid, Spain; 2 Unidad de Enfermedades Infecciosas/VIH; Hospital General Universitario “Gregorio Marañón”, Madrid, Spain; 3 Instituto de Investigación Sanitaria Gregorio Marañón (IiSGM), Madrid, Spain

## Abstract

The mechanisms involved in the chronic hepatitis C progression are incompletely understood. The aim was to analyze the association between *2*′*5*′*oligoadenylate synthetase 1,2 and 3 (OAS1-3*) and *myxovirus resistance proteins 1 (Mx1*) polymorphisms and severity of liver disease in human immunodeficiency virus (HIV)/hepatitis C virus (HCV) coinfected patients. We performed a cross-sectional study in 219 patients that underwent a liver biopsy. DNA genotyping for *Mx1* (rs469390), *OAS1* (rs2285934), *OAS2* (rs1293762) and *OAS3* (rs2010604) was performed by using GoldenGate assay. The outcome variables ion liver biopsy were: (i) significant fibrosis (F ≥ 2); (ii) moderate activity grade (A ≥ 2). Additive model of inheritance for genetic association test was used. The likelihood of having significant fibrosis (F ≥ 2) was lower in patients carrying *OAS2* rs1293762 A allele [adjusted odds ratio (aOR) = 0.51; p = 0.040]. Besides, the likelihood of having moderate activity grade (A ≥ 2) was higher in patients carrying *Mx1* rs464397 C allele (aOR = 1.63; p = 0.028) and *Mx1* rs469390 G allele (aOR = 1.97; p = 0.005), while it was lower in patients carrying *OAS1* rs2285934 A allele (aOR = 0.64; p = 0.039) and *OAS2* rs1293762 A allele (aOR = 0.41; p = 0.009). In conclusion, *Mx1* and *OAS1-2* polymorphisms were associated with the severity of liver disease in HIV/HCV-coinfected patients, suggesting a significant role in the progression of hepatic fibrosis.

Only a minority of patients may spontaneously clear the hepatitis C virus (HCV) during the acute phase of infection, which correlates with a rapid induction of innate response, especially interferon-stimulated genes (ISGs)^1^. However, the majority of patients is unable to clear the HCV and develops chronic hepatitis C (CHC), which may cause irreversible fibrosis, leading to cirrhosis and hepatocellular carcinoma^1^,^2^. Moreover, CHC is accelerated in human immunodeficiency virus (HIV)/HCV coinfected patients, resulting in higher rates of disease progression than HCV mono-infected patients^3^.

The pathogenic mechanisms involved in this progress are incompletely understood, but it is known that the alternations of liver inflammatory profiles and the impaired immune responses are thought to be an important determinant of the rate of fibrosis progression^2^. Among others, chronic HCV infection has a pronounced effect on gene expression of a subset of ISGs such as 2′5′oligoadenylate synthetase (OAS) family and myxovirus resistance proteins (Mx)^4^. The Mx1 protein is a GTPase that acts at an early step of the virus life cycle, prior to the genome replication, by trapping viral components and preventing them from reaching their cellular destination^4^. OAS proteins are able to synthesize 2′,5′-oligomers, which specifically activate the latent form of RNaseL, which may then mediate RNA degradation, contributing to decay of viral RNA^4^. Single nucleotide polymorphisms (SNPs) in the *OAS* gene (chromosome 12) and *Mx* gene (chromosome 21) have been related to severity and/or outcome of viral infections such as tick-borne encephalitis^5^, West Nile virus^6^, dengue virus^7^, coronavirus^8^, and hepatitis B^9^. Regarding to HCV infection, *Mx1* rs2071430 polymorphism has been related to HCV clearance during acute HCV infection and after interferon (IFN) therapy, and higher severity of liver disease in HCV-monoinfected patients^10^,^11^,^12^,^13^. However, to our knowledge, there is no report related to *OAS/Mx* SNPs and severity of liver disease in HIV/HCV coinfected patients.

The aim of this study was to analyze the association between *Mx1* and *OAS1-3* polymorphisms and severity of liver disease in HIV/HCV coinfected patients.

## Results

### Characteristics of the study population

Table 1 shows the epidemiological and clinical characteristics of 219 HIV/HCV-coinfected patients at the time of liver biopsy. Regarding liver biopsy, 49.8% had significant fibrosis (F ≥ 2), 52.6% had moderate necroinflammatory activity (A ≥ 2), and 15.3% had alanine aminotransferase (ALT) ≥ 150 IU/L.

### *Mx1* and *OAS1-3* polymorphisms

Table 2 shows the characteristics of SNPs, which displayed missing values < 5% and were in HWE (p > 0.05). When SNPs frequencies in HIV/HCV coinfected patients were compared to the SNPs frequencies in healthy subjects from the IBS, we only found differences between groups for *Mx1* rs469390 AA (p = 0.011) and *Mx1* rs469390 AG (p = 0.036) genotypes.

### Association between *Mx1* and *OAS1-3* polymorphisms and liver disease

Table 3 shows the relationship of *Mx1* and *OAS1-3* polymorphisms with severity of liver disease under a model of additive inheritance, which was the genetic model that best fitted our data. The trend of having significant fibrosis (F ≥ 2) was lower in patients carrying of *OAS2* rs1293762 A allele (p = 0.023). The trend of having moderate activity grade (A ≥ 2) was higher in patients carrying *Mx1* rs464397 C allele (p = 0.015) and *Mx1* rs469390 G allele (p = 0.003); while it was lower in patients carrying *OAS1* rs2285934 A allele (p = 0.026), *OAS2* rs1293762 A allele (p = 0.002), and it was nearly significant for *OAS3* rs2010604 C allele (p = 0.095). Moreover, the likelihood of having significant fibrosis (F ≥ 2) in liver biopsy was lower in patients carrying *OAS2* rs1293762 A allele [adjusted odds ratio (aOR) = 0.51; p = 0.040]. Besides, the likelihood of having moderate activity grade (A ≥ 2) was higher in patients carrying *Mx1* rs464397 C allele (aOR = 1.63; p = 0.028) and *Mx1* rs469390 G allele (aOR = 1.97; p = 0.005), while it was lower in patients carrying *OAS1* rs2285934 A allele (aOR = 0.64; p = 0.039) and *OAS2* rs1293762 A allele (aOR = 0.41; p = 0.009).

**Figure 1:**
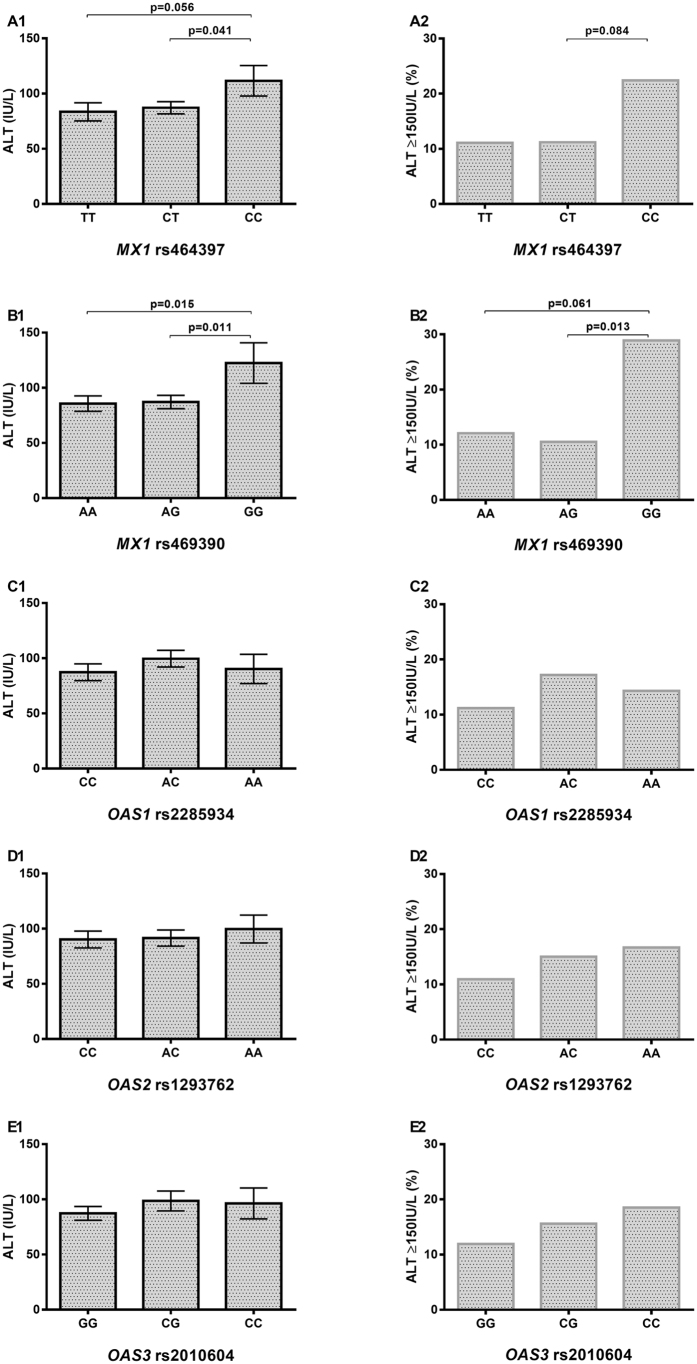
Summary of the differences of serum ALT according to genotypes of *Mx1* rs464397, *MX1* rs469390, OAS1 rs2285934, OAS2 rs1293762 and OAS3 rs2010604 in HIV/HCV coinfected patients. P-values were calculated by Student’s t-test and Chi-squared test. Abbreviations: OAS, 2′5′oligoadenylate synthetase; Mx1, myxovirus resistance proteins.

Figure 1 shows the relation between *MX1* and *OAS1-3* genetic polymorphisms and serum ALT. We found that patients carrying *Mx1* rs464397 CC genotype and *Mx1* rs469390 GG genotype had higher values of serum ALT and percentage of patients with ALT ≥ 150 IU/L. However, we did not find any significant association in multivariate regression analysis (*data not shown*). Moreover, we also analyzed the relation between *MX1* and *OAS1-3* polymorphisms and serum aspartate aminotransferase (AST), but we did not find any association (*data not shown*).

Table 4 shows the relationship among haplotype of *Mx1* gene (rs464397, rs458582, rs469390) and *OAS1-3* cluster (rs2285934, rs2285933, rs2010604, rs739903, rs1293762) and severity of liver disease. The likelihood of having moderate activity grade (A ≥ 2) in liver biopsy was higher in patients with *Mx1* CTG haplotype (aOR = 1.44; p < 0.001) and lower in patients with *Mx1* CTA haplotype (aOR = 0.62; p = 0.007). Moreover, the likelihood of having moderate activity grade (A ≥ 2) in liver biopsy was lower in patients with *OAS1-3* AGCTA haplotype (aOR = 0.78; p = 0.008).

## Discussion

To our knowledge, this study is the first description of the relationship between *Mx1* and *OAS1-3* polymorphisms and severity of liver disease in HIV/HCV-coinfected patients. The major findings of the present study were that *Mx1* rs464397 C allele, *Mx1* rs469390 G allele, and *Mx1* CTG haplotype were associated with higher odds of having moderate activity grade (A ≥ 2) in liver biopsy, while *OAS1* rs2285934 A allele, *OAS2* rs1293762 A allele, and *OAS1-3* AGCTA haplotype were related to lower likelihood of having moderate activity grade (A ≥ 2). Besides, *OAS2* rs1293762 A allele was related to lower likelihood of having significant fibrosis (F ≥ 2) in liver biopsy. Thus, *Mx1* and *OAS1-2* polymorphisms might play a role in developing of liver disease in HIV/HCV-coinfected patients.

In our study, we included a control group to show that our study cohort had allelic and genotypic frequencies similar to the general population. However, we found significant differences in allelic and genotypic frequencies of *Mx1* rs469390 between HIV/HCV coinfected patients and Iberian populations in Spain from HapMap data. This observation might suggest that *Mx1* rs469390 could be associated to innate resistance to HCV infection. However, our study design is not adequate to draw such conclusions due to a number of factors that we do not control. For example, our cohort may have a survival bias because patients were more than 20 years of median with HIV and HCV infections. In addition, patients were selected due to they were candidates for antiviral treatment against HCV because progression of liver disease was suspected.

In the setting of CHC, the immune system initially attempts to eradicate the HCV, but besides, probably promotes hepatocyte damage and fibrosis through direct cellular toxicity and the release of inflammatory cytokines^2^. The innate immune response against viruses produce type I IFN (alpha, beta), which induces the expression of Mx1 protein^4^,^14^. Although its effect against HCV is not well documented, high levels of Mx1 protein have been detected in IFN-treated HCV patients with sustained virological response^15^. In fact, *Mx1* rs2071430 polymorphism, in the promoter region, has been related to *Mx1* gene expression, spontaneous viral clearance during acute HCV infection, higher hepatic fibrosis score and hepatitis activity grade, and response to IFN therapy in HCV-monoinfected patients^10^,^11^,^12^,^13^. In the *in silico* analysis performed, we found that *Mx1* rs464397 seems to be implicated in both proximal (open chromatin region, transcription factor binding sites and histone markers) and distal regulation. Furthermore, rs464397 seems also be involved in RNA binding protein-mediated post-transcriptional regulation as well as to be a SNP with experimental expression quantitative trait loci (eQTL) evidence. On the other hand, *Mx1* rs469390 polymorphism is a missense variant (V379I) that might not affect protein structure; but it seems influence an exonic splicing enhancer motif and it might be involved in protein domain abolishing. In this way, *MX1* rs469390 polymorphism could decrease expression of MX1, getting worse the control of HCV, and might therefore be related to the increase of sustained inflammation and progression of liver disease.

The OAS proteins play an important role in the innate immune response and genetic variants in the *OAS* genes are known to affect OAS activity and are associated with outcome of viral infections^16^. The *OAS1* rs2660, rs10774671, and rs3741981 polymorphisms have been related to persistent HCV infection^11^,^17^ and progression of HCV disease^18^; and *OAS1* polymorphism at exon 7 splice acceptor site with response to IFN therapy in HCV infected patients^19^. The *OAS2* polymorphism rs1293762 has been associated with predisposition to CHC^20^. In our study, *OAS1-3* polymorphisms were related to lower likelihood of having significant liver disease (A ≥ 2 and/or F ≥ 2), indicating that the A-allele conferred a protective effect with respect to liver disease progression. *OAS1* rs2285934 and *OAS2* rs1293762 polymorphisms are located in intron 3 and intron 2, respectively. In the *in silico* analysis, both SNPs seem to be involved in RNA binding protein-mediated post-transcriptional gene regulation. In this way, *OAS1* rs2285934 and *OAS2* rs1293762 polymorphisms could increase expression of OAS1 and OAS2, improving the control of HCV, and might therefore be related to the decrease of sustained inflammation and progression of liver disease.

As discussed above, the presence of *Mx1* and *OAS1-3* polymorphisms may be related to have moderate or significant liver disease. This association that we observed might be due to the proximity to other SNPs that may modulate *Mx1* gene expression, or a direct effect of *Mx1* and *OAS1-3* polymorphisms analyzed in this study. On the one hand, haplotype analysis showed a stronger association with liver disease than that found with each polymorphism alone. On the other hand, the *in silico* analysis showed that polymorphisms alone could have some influence on the expression of the proteins involved. However, it would be necessary to demonstrate if these *Mx1* and *OAS1-3* polymorphisms may modify the expression of *Mx1* and *OAS1-3* genes.

The impact of *OAS/MX1* SNPs on fibrosis progression in CHC has not been picked up in the GWAS previously carried out^21^,^22^. However, we found a relatively strong association between *OAS/MX1* SNPs and the severity of liver disease in our HIV/HCV coinfected patients. This could be because: (a) GWAS usually does not have the power to detect all, only the biggest effects. Besides, note that strong statistical adjustments on the p-value are carried out for correcting multiple testing; (b) we studied a SNP candidate with MAF greater than 20%. In these cases, an individual effect is more easily detected when the size of sample is small^23^; (c) we should not discard the effect of HIV infection on the immune system, which could show the influence of *OAS/MX1* polymorphisms on CHC progression.

Finally, several aspects have to be taken into account for the correct interpretation of the results. Firstly, this is a cross-sectional study with a limited number of patients, which could limit achieving statistically significant values. Besides, the cross-sectional design nature of the analyses may be a confounding factor. Secondly, data were collected retrospectively, which entails a lack of uniformity. Thirdly, all selected patients met a set of criteria for starting HCV treatment (e.g., no alcohol abuse, high CD4 cell counts, controlled HIV replication, and good treatment adherence), and this may have introduced a selection bias. Fourthly, regarding the statistical significance, there is a considerable controversy about adjusting the “p-value” after multiple tests on clinical-orientated studies^24^,^25^. In our study, there was a hypothesis supported by theory and previous reports that related *Mx1* and *OAS1-3* polymorphisms to severity and/or outcome of viral infections^5^,^6^,^7^,^8^,^9^, including HCV infection^10^,^11^,^12^,^13^. Therefore, we were not literally doing a random search of a meaningful result, and our results should not be affected by the fact of carrying out a high number of statistical tests. Fifthly, this study was performed on patients with European ancestry, and it would be interesting to perform these analyses on different ethnic groups. Finally, our study only included HIV/HCV-coinfected patients and it would be interesting to know the role of studied polymorphisms in HCV monoinfected patients, but we did not have access to a cohort of HCV monoinfected patients.

In conclusion, *Mx1* and *OAS1-2* polymorphisms were associated with the severity of liver disease in patients coinfected with HIV and HCV, suggesting that these polymorphisms might play a significant role in the progression of hepatic fibrosis. Further analysis involving large numbers of patients are needed to corroborate these associations that we have found.

## Materials and Methods

### Study population

We performed a cross-sectional study in 219 HIV/HCV-coinfected patients that underwent a liver biopsy between September 2000 and November 2008. The study was conducted in accordance with the Declaration of Helsinki and patients gave their written consent for the study. The Institutional Review Board and the Research Ethic Committee of the Instituto de Salud Carlos III approved the study. Each participating patient signed an informed consent form.

Liver biopsies were performed on patients who were potential candidates for anti-HCV therapy and had not received previous IFN therapy (naïve for HCV-treatment). Selection criteria were: no clinical evidence of hepatic decompensation, detectable HCV RNA by polymerase chain reaction (PCR), negative hepatitis B surface antigen, availability of DNA sample, CD4 + lymphocyte count higher than 200 cells/μL, and stable combination antiretroviral therapy (cART) for at least 6 months before study entry or no need for cART according to treatment guidelines used in the study period^26^,^27^. Patients with active opportunistic infections, active drug addiction, and other concomitant severe diseases were excluded. All subjects included in our study were European white.

### Epidemiological and clinical data

Clinical and epidemiological data were obtained from medical records. The duration of HCV infection for patients with a history of intravenous drug use was estimated starting from the first year they shared needles and other injection paraphernalia, which are the most relevant risk practices for HCV transmission^28^. The duration of HCV infection was not calculated when the date of initiation of their HCV infection could not be determined with certainty. Consumption of more than 50 g of alcohol per day for at least 12 months was considered as a high intake. Body mass index (BMI) was calculated as the weight in kilograms divided by the square of the height in meters.

Biochemistry panel was measured using an autoanalyzer Hitachi 912 (Boehringer Mannheim, Germany), while patients were fasting. The degree of insulin resistance was estimated for each patient using the homeostatic model assessment (HOMA)^29^: fasting plasma glucose (mmol/L) times fasting serum insulin (mU/L) divided by 22.5.

### HCV assays

HCV infection was documented in all patients by enzyme-linked immunosorbent assay and PCR test. HCV genotype was determined by hybridization of biotin-labeled PCR products to oligonucleotide probes bound to nitrocellulose membrane strips (INNO-LiPA HCV II, Innogenetics, Ghent, Belgium). Plasma HCV-RNA viral load was measured by PCR (Cobas Amplicor HCV Monitor Test, Branchburg, NJ, USA) and real-time PCR (COBAS AmpliPrep/COBAS TaqMan HCV test); and results were reported in terms of international units per milliliter (IU/mL). The limit of detection varied between 10 IU/mL and 600 IU/mL depending on the HCV diagnostic test used at the time of biopsy. There was no patient with HCV viral loads below the limit of detection. The HCV RNA level of 500,000 UI/ml was chosen as cutoff for low vs. high viral load (circulating HCV RNA)^30^,^31^.

### Liver biopsy

Liver biopsies were performed as we described previously^32^. The samples were always evaluated by the same pathologist, who was unaware of the patients’ clinical or laboratory data. Liver fibrosis and necroinflammatory activity were estimated according to Metavir score as follows^33^: F0, no fibrosis; F1, mild fibrosis; F2, significant fibrosis; F3, advanced fibrosis; and F4, definite cirrhosis. The degree of necroinflammation (activity grade) was scored as follows: A0, no activity; A1, mild activity; A2, moderate activity; A3, severe activity.

### Genotyping of DNA polymorphisms

**Figure 2:**
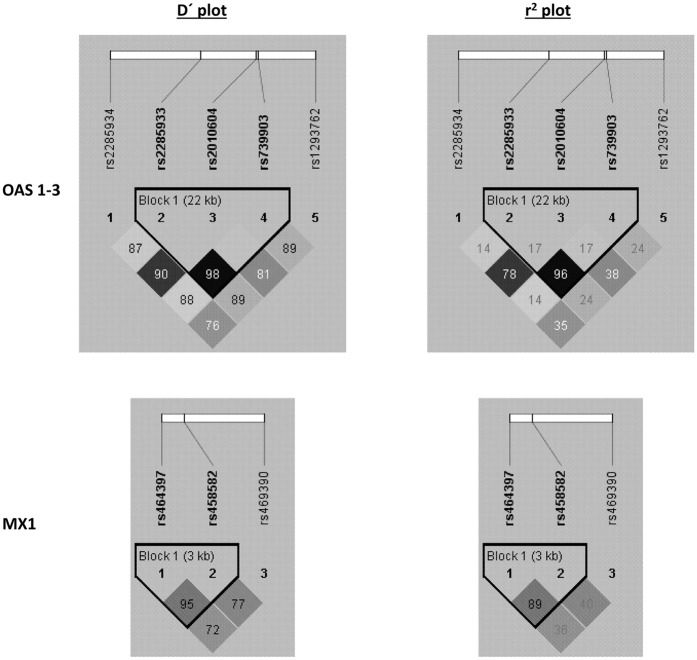
Pairwise linkage disequilibrium (LD) patterns for genetic polymorphisms of *MX1* and *OAS1-3*. Each diagonal represents a different SNP, with each square representing a pairwise comparison between two SNPs. Abbreviations: OAS, 2′5′oligoadenylate synthetase; Mx1, myxovirus resistance proteins.

The most common SNPs in *Mx1* gene (chromosome 21) and *OAS1-3* genes (chromosome 12) were selected using the databases of HapMap Project (http://snp.cshl.org/cgi-perl/gbrowse/hapmap_B35/) and NCBI (dbSNP) (http://www.ncbi.nlm.nih.gov/entrez/). The selection criteria were: (i) SNPs located in putative regulatory regions, or splicing control elements (SCE), or missense variants; (ii) minor allelic frequency (MAF) greater than 20% in European people in order to have guarantees to obtain significant associations when working with small sample sizes^34^. With this setting, we found three SNPs in *Mx1* gene (rs464397 in intron 3, rs458582 in intron 5, and rs469390 in exon 13 (missense)) and other five SNPs at *OAS* genes (*OAS1* rs2285934 in intron 3, *OAS2* rs1293762 in intron 2, *OAS3* rs739903 in 3′UTR, *OAS3* rs2285933 in exon 6 (missense), and *OAS3* rs2010604 in 3′UTR). Finally, five polymorphisms were selected as tagSNPs according to linkage disequilibrium (LD) (Fig. 2): two SNPs at *Mx1* (rs464397 and rs469390) and three SNPs at *OAS (OAS1* rs2285934, *OAS2* rs1293762, and *OAS3* rs739903). Three SNPs (*Mx1* rs458582, *OAS3* rs2285933, and *OAS3* rs2010604) were discarded for the analysis of individual association because these SNPs were at high linkage disequilibrium (LD) (Fig. 2).

Genomic DNA was extracted from peripheral blood with Qiagen kit (QIAamp DNA Blood Midi/Maxi; Qiagen, Hilden, Germany). DNA samples were sent at the Spanish National Genotyping Center (CeGen; http://www.cegen.org/) for DNA genotyping by using GoldenGate assay with VeraCode Technology (Illumina Inc., San Diego, California, USA).

Moreover, for healthy subjects, the frequencies of alleles and genotypes for studied polymorphisms were obtained using the 1000 Genomes Project website (http://www.1000genomes.org/home), which provide a broad representation of common human genetic variation by applying whole-genome sequencing to a diverse set of individuals from multiple populations^35^. We select the IBS (Iberian populations in Spain) population that included 107 individuals.

### Outcome variables

The primary outcome variables were related to severity of liver disease: significant fibrosis (F ≥ 2) and moderate activity grade (A ≥ 2). These outcomes were developed after a minimum follow-up time of 10 years with HCV infection.

### Statistical analysis

Chi-square test, Chi-square test for trend (linear by linear association), Student’s t-test, and multivariate logistic regression analysis were used to investigate the relationship among *Mx1* and *OAS* polymorphisms and severe liver disease. We included the SNP with the Enter algorithm (Forced Entry) and the covariables with the Stepwise algorithm (at each step, factors are considered for removal or entry: a p-value for entry and exit of 0.15 and 0.20, respectively). Thus, each logistic regression test was only adjusted by the most significant co-variables associated with each one of the outcome variables, avoiding the over-fitting of the regression. The covariables used were gender, age, alcohol intake at biopsy study, BMI, HOMA, nadir CD4+ T-cells, AIDS, undetectable HIV-RNA (<50 copies/ml), CD4+ T-cells, time on cART, type of cART, HCV-RNA ≥500,000 IU/ml, time of HCV infection, and HCV genotype. Besides, we also used data of the *PNPLA3* rs738409 polymorphism and the *IL28B* rs12980275 polymorphism, which have already been published by us and showed significant association with liver fibrosis in HIV/HCV coinfected patients^36^,^37^. The genetic association study was carried out according to the genetic model that best fit our data (additive, recessive, dominant, codominant, and overdominant). These analyses were tested according to the goodness of fit evaluated by Akaike information criterion (AIC) value and Bayesian information criterion (BIC). These analyses were performed by using the IBM SPSS Statistics for Windows, Version 21.0 (IBM Corp, Chicago, Armonk, NY, USA).

Hardy-Weinberg equilibrium (HWE) for all SNPs was assessed by a Chi-square test, considering equilibrium when p > 0.05. In addition, pair-wise linkage disequilibrium (LD) analysis was computed to detect the inter-marker relationship using the standardized D′ and r^2^ values by Haploview 4.2 software. Haplotype-based association testing was performed using Plink software (http://pngu.mgh.harvard.edu/~purcell/plink/) comparing each haplotype with the rest of haplotypes (there was no reference category). All p-values were two-tailed and statistical significance was defined as p < 0.05.

The *in silico* analysis for possible functional implication of each polymorphism was evaluated by using two web-tools: a) VarioWatch (http://genepipe.ncgm.sinica.edu.tw/variowatch/); b) rSNABase (http://rsnp.psych.ac.cn/).

## Additional Information

**How to cite this article**: García-Álvarez, M. *et al. Mx1, OAS1* and *OAS2* polymorphisms are associated with the severity of liver disease in HIV/HCV-coinfected patients: A cross-sectional study. *Sci. Rep.* **7**, 41516; doi: 10.1038/srep41516 (2017).

**Publisher's note:** Springer Nature remains neutral with regard to jurisdictional claims in published maps and institutional affiliations.

## Figures and Tables

**Table 1:** Clinical and epidemiological characteristics of HIV/HCV-coinfected patients.

Characteristics	All Patients
**No.**	219
**Male**	162 (74%)
**Age, years**	39.8 (37.4; 44)
**Epidemiological history**	
**HIV acquired by IVDU**	193 (88.1%)
**Years since HCV infection**	21.3 (17; 24.3)
**High alcohol intake**	119 (54.3%)
**High alcohol intake at biopsy study**	30 (13.7%)
**CDC category C**	60 (27.4%)
**Metabolic markers**	
**BMI, kg/m^2^**	22.3 (20.8; 24.6)
BMI ≥ 25 kg/m^2^	50 (23%)
**HOMA**	2.10 (1.27; 3.75)
HOMA ≥ 3	71 (33.5%)
**Antiretroviral therapy**	
**cART**	183 (83.6%)
**Time on cART, years**	4.4 (2.5; 6.6)
**Current cART protocols**	
Non-treated	36 (16.4%)
PI-based	50 (22.8%)
NNRTI-based	114 (52.1%)
NRTI-based	19 (8.7%)
**HIV markers**	
Nadir CD4+, T cells/μL	189 (83; 315)
CD4+, T cells/μL	467 (324; 672)
HIV-RNA < 50 copies/mL	162 (74%)
**HCV markers**	
**HCV genotype** (n = 215)	
1	122 (56.7%)
2	5 (2.3%)
3	50 (23.3%)
4	38 (17.7%)
**HCV RNA > 500,000 UI/mL** (n = 216)	161 (74.5%)
**Liver biopsy (Metavir score)**	
**Hepatic fibrosis**	
F0	25 (11.4%)
F1	85 (38.8%)
F2	60 (27.4%)
F3	26 (11.9%)
F4	23 (10.3%)
**Activity grade** (n = 215)	
A ≤ 1	102 (47.5%)
A2	89 (41.4%)
A3	24 (11.2%)
**Aminotransferases**	
ALT (IU/L) (n = 219)	72 (45; 114)
ALT ≥ 150 IU/L	33 (15.3%)
AST (IU/L) (n = 215)	55 (37; 82)
AST ≥ 100 IU/L	33 (15.3%)
AST: ALT (n = 215)	0.79 (0.63; 0.97)
AST: ALT < 1	110 (50.2%)

Values are expressed as absolute numbers (%) and median (percentile 25; percentile 75). Sometimes, the percentages were not calculated from all patients because some data was missing.

Abbreviations: BMI, body mass index; HOMA, homeostatic model assessment; HCV, Hepatitis C virus; CDC category C, centers for disease control and prevention classification system, clinical category C which includes any condition listed in the CDC’s 1897 surveillance case definition of AIDS; PI-based, protein inhibitor-based therapy; NNRTI-based, non-nucleoside reverse transcriptase inhibitor -based therapy; 3NRTI-based, triple nucleoside regimen; HIV; Human immunodeficiency virus; HIV-RNA, HIV plasma viral load; HCV-RNA, HCV plasma viral load; ALT, alanine aminotransferase; AST Aspartate Aminotransferase.

**Table 2:** Summary of Hardy Weinberg Equilibrium and frequencies of alleles and genotypes for *Mx1* and *OAS1-3* polymorphisms in HIV/HCV coinfected patients compared to Iberian populations in Spain from HapMap data (http://www.ncbi.nlm.nih.gov/bioproject/59819).

SNPs	HWE	HIV/HCV group (n = 219)				IBS group (n = 107)				χ^2^ test(a)	χ^2^ test (b)
	p-value	Alleles		Genotype		Alleles		Genotype		p-value	p-value
***Mx1*****rs464397**	0.420	C	53%	CC	26%	C	53%	CC	28%	0.906	0.802
		T	47%	CT	53%	T	47%	CT	50%		0.695
				TT	21%			TT	22%		0.949
***Mx1*** **rs469390**	0.410	A	56%	AA	30%	A	64%	AA	45%	0.208	**0.011**
		G	44%	AG	52%	G	35%	AG	39%		**0.036**
		—	—	GG	17%	—	—	GG	16%		0.945
***OAS1*** **rs2285934**	0.450	C	66%	CC	45%	C	67%	CC	41%	0.956	0.572
		A	34%	CA	42%	A	33%	CA	51%		0.157
		—	—	AA	13%	—	—	AA	8%		0.250
***OAS2*** **rs1293762**	0.790	C	54%	CC	29%	C	52%	CC	25%	0.824	0.531
		A	46%	CA	49%	A	48%	CA	54%		0.465
		—	—	AA	22%	—	—	AA	21%		0.950
**OAS3 rs2010604**	0.360	G	67%	GG	46%	G	66%	GG	42%	0.955	0.573
		C	33%	GC	41%	C	33%	GC	49%		0.212
		—	—	CC	12%	—	—	CC	8%		0.364

Statistically significant differences are shown in bold. P-values were calculated by Chi-squared test; (a), differences between allele frequencies; (b), differences between genotype frequencies.

Abbreviations: HWE, Hardy Weinberg Equilibrium; HIV, human immunodeficiency virus; HCV, hepatitis C virus; IBS, Iberian populations in Spain; OAS, 2′5′oligoadenylate synthetase; Mx1, myxovirus resistance proteins.

**Table 3:** Summary of the differences of genotypic frequencies (*Mx1* rs464397, *MX1* rs469390, OAS1 rs2285934, OAS2 rs1293762 and OAS3 rs2010604) in relation to severity of liver disease and the adjusted logistic regression analysis for the polymorphisms in HIV/HCV coinfected patients.

SNPs	Unadjusted				Adjusted	
***Mx1*****rs464397**	**TT**	**CT**	**CC**	**P**^(**a**)^	**aOR (95%CI)**	**p**^(**b**)^
F ≥ 2	42.2% (19/45)	50.0% (58/116)	55.2% (32/58)	0.198	1.15 (0.75; 1.77)	0.501
A ≥ 2	38.6% (17/44)	52.6% (60/114)	63.2% (36/57)	0.015	1.63 (1.05; 2.52)	0.028
***Mx1*****rs469390**	**AA**	**AG**	**GG**	**P**^(**a**)^	**aOR (95%CI)**	**p**^(**b**)^
F ≥ 2	39.4% (26/66)	53.5% (61/114)	55.3% (21/38)	0.077	1.22 (0.80; 1.88)	0.347
A ≥ 2	40% (25/65)	53.6% (60/112)	70.3% (26/37)	0.003	1.97 (1.23; 3.16)	0.005
***OAS1*****rs2285934**	**CC**	**AC**	**AA**	**P**^(**a**)^	**aOR (95%CI)**	**p**^(**b**)^
F ≥ 2	52.0% (51/98)	49.5% (46/93)	42.9% (12/28)	0.415	0.88 (0.58; 1.33)	0.539
A ≥ 2	60.4% (58/96)	48.4% (44/91)	39.3% (11/28)	0.026	0.64 (0.42; 0.98)	0.039
***OAS2*****rs1293762**	**CC**	**AC**	**AA**	**P**^(**a**)^	**aOR** (**95%CI**)	**p**^(**b**)^
F ≥ 2	60.9% (39/64)	47.7% (51/107)	39.6% (19/48)	0.023	0.51 (0.26; 0.97)	0.040
A ≥ 2	66.7% (42/63)	51.0% (53/104)	37.5% (18/48)	0.002	0.41 (0.21; 0.80)	0.009
***OAS3*****rs2010604**	**GG**	**CG**	**CC**	**P**^(**a**)^	**aOR (95%CI)**	**p**^(**b**)^
F ≥ 2	52.5% (53/101)	48.9% (44/90)	44.4% (12/27)	0.431	0.91 (0.59; 1.38)	0.651
A ≥ 2	59% (59/100)	48.3% (42/87)	44.4% (12/27)	0.095	0.74 (0.48; 1.13)	0.169

Statistically significant differences are shown in bold. ^(a)^P-values were calculated by Chi-squared test for trend (linear by linear association); ^(b)^P-values were calculated by multivariate logistic regression adjusted by the most important clinical and epidemiological characteristics (see **statistical analysis** section). Sometimes, the percentages were not calculated from all patients because some data was missing.

Abbreviations: 95%CI, 95% of confidence interval; aOR, adjusted odds ratio; p-value, level of significance; A ≥ 2, moderate activity (Metavir); F ≥ 2, significant fibrosis (Metavir); OAS, 2′5′oligoadenylate synthetase; Mx1, myxovirus resistance proteins.

**Table 4:** Association of haplotype of *Mx1* gene and *OAS1-3* cluster with severity of liver disease in HIV/HCV coinfected patients.

					F ≥ 2			A ≥ 2	
					Freq.	aOR (95% CI)	p	aOR (95% CI)	p
***Mx1*****haplotypes**									
**rs464397**	**rs458582**	**rs469390**							
C	T	G			0.377	1.44 (0.92–2.26)	0.109	2.38 (1.45–3.91)	**<0.001**
T	G	A			0.413	0.85 (0.54–1.34)	0.487	0.68 (0.42–1.09)	0.104
C	T	A			0.141	0.62 (0.32–1.22)	0.165	0.39 (0.19–0.79)	**0.007**
***OAS1-3*** **haplotypes**									
**rs2285934**	**rs2285933**	**rs2010604**	**rs739903**	**rs1293762**					
A	G	C	T	A	0.281	0.78 (0.50–1.21)	0.261	0.55 (0.35–0.87)	**0.008**
C	G	G	T	A	0.133	0.66 (0.34–1.25)	0.195	0.93 (0.50–1.76)	0.836
C	C	G	C	C	0.239	1.14 (0.70–1.84)	0.592	1.40 (0.85–2.29)	0.181
C	G	G	T	C	0.250	1.33 (0.82–2.16)	0.250	1.45 (0.88–2.39)	0.140

Statistically significant differences are shown in bold. P-values were calculated by multivariate logistic regression adjusted by the most important clinical and epidemiological characteristics (see **statistical analysis** section). Haplotypes with frequencies less than 5% have not included.

Abbreviations: aOR, adjusted odds ratio; 95%CI, 95% of confidence interval; p-value, level of significance; A ≥ 2, moderate activity (Metavir); F ≥ 2, significant fibrosis (Metavir); OAS, 2′5′oligoadenylate synthetase; Mx1, myxovirus resistance proteins.
